# Association of Body Roundness Index with all-cause and cardiovascular mortality in patients with cardiovascular-kidney-metabolic (CKM) syndrome stages 0–3: a cohort study

**DOI:** 10.1016/j.ahjo.2026.100779

**Published:** 2026-04-04

**Authors:** Zehua He, Wenjing Xiong, Yajing Li, Xia Wu, Yiyun Zhang, Xiaoyun Shan, Yan Liu, Weiqing Rang

**Affiliations:** a School of Public Health, University of South China, Hengyang, 421001, China; b China Institute for History of Medicine and Medical Literature, China Academy of Chinese Medical Sciences, No.16, South Small Street, Dongzhimen, Dongcheng District, Beijing, 100700, China

**Keywords:** Body roundness index (BRI), Cardiovascular-kidney-metabolic (CKM) syndrome, Mortality

## Abstract

**Objective:**

This study aimed to investigate the association of body roundness index (BRI) with all-cause and cardiovascular mortality in patients with cardiovascular-kidney-metabolic (CKM) syndrome stages 0–3.

**Methods:**

We analyzed data from 18,984 participants in the National Health and Nutrition Examination Survey (NHANES) from 2003 to 2018. We employed Cox proportional hazards models, Fine-Gray proportional subdistribution hazards models, restricted cubic splines (RCS), and subgroup analysis to examine the associations between BRI and all-cause and cardiovascular mortality in patients with CKM syndrome stages 0–3.

**Results:**

Over a median follow-up of 7.5 years, 1756 all-cause and 471 cardiovascular deaths were documented. Cox and Fine-Gray models revealed a significant positive association between the BRI and both all-cause and cardiovascular mortality after full adjustment. RCS indicated a U-shaped nonlinear relationship between BRI and all-cause mortality (*P* for nonlinear <0.0001), while a linear association was observed with cardiovascular mortality (*P* for nonlinear = 0.0635). Threshold analysis identified an inflection point at BRI = 5.68. BRI levels below 5.68 showed a negative correlation (HR = 0.90, 95% *CI*: 0.83–0.96, *P* = 0.0024); above 5.68 BRI was positively correlated (HR = 1.12, 95% *CI*: 1.08–1.17, *P* < 0.0001). Subgroup analyses revealed significant interactions (*P* for interaction <0.05) whereby the association was strongest among participants with less than high school diploma and current drinkers (for all-cause mortality), and among younger participants(< 50 years) and current smokers (for cardiovascular mortality).

**Conclusion:**

In CKM syndrome stages 0–3, BRI shows a U-shaped link to all-cause mortality but a linear association with cardiovascular mortality.

## 1 Introduction

In 2023, cardiovascular-kidney-metabolic (CKM) syndrome gained significant attention as an integrative concept that emphasizes the close pathophysiological interconnections among obesity, type 2 diabetes, chronic kidney disease(CKD), and cardiovascular disease(CVD) [1]. A Study has indicated that from 2011 to 2020, approximately 90.8% of the population met the criteria for stages 0–3 of CKM syndrome [2], [3]. Furthermore, data from 2015 to 2020 showed that more than a quarter of the U.S. population may have CKM syndrome [4]. The significant impact stemming from the high prevalence and mortality rates of CKM syndrome imposes a substantial burden on society [1], [5]. The American Heart Association (AHA) introduced a staging system (Stages 0–4) for CKM syndrome in 2023, providing a new framework for risk assessment and clinical intervention [1]. Meanwhile, the AHA has underscored that preclinical prediction is imperative for individual care and recommends that research on populations with stages 0–3 CKM syndrome should prioritize the prevention of CVD events [5], [6].

Adipose dysfunction, particularly visceral fat accumulation, is recognized as a central mechanism in the development and progression of CKM syndrome [7]. This underscores the importance of assessing body fat distribution. Conventional indices such as Body Mass Index (BMI) are limited in accurately capturing body fat distribution [8], [9]. Compared to other anthropometric measures like waist circumference (WC), waist-to-hip ratio (WHR), or A Body Shape Index (ABSI) which focus on linear or ratio-based assessments, the BRI, introduced by Thomas et al. in 2013, is based on an elliptical geometric model that incorporates WC and height, enabling a more accurate estimation of visceral and total adiposity [10]. Numerous studies have since linked BRI to conditions such as diabetes [11], CKD [12], metabolic syndrome(MetS) [13], and cardiovascular events [14]. Recent studies have shown that BRI outperforms waist circumference (WC), waist-to-hip ratio (WHR), and ABSI in predicting visceral adipose tissue area and metabolic risk in both general and clinical populations [15], [16].

However, the prognostic value of BRI specifically within the integrated framework of stages 0–3 CKM syndrome remains insufficiently investigated, particularly for hard endpoints such as all-cause and cardiovascular mortality. While BRI is predictive of individual cardiometabolic conditions, its stage-dependent association with mortality remains unelucidated. While Cai et al. observed that the predictive utility of BRI for cardiovascular disease attenuates in stage 3 [17], it is unknown whether this pattern extends to mortality outcomes or differs between all-cause and cardiovascular mortality. Therefore, using data from the National Health and Nutrition Examination Survey (NHANES 2003–2018), this study aims to evaluate the relationship between BRI and mortality risk in stages 0–3 CKM syndrome, with the goal of improving risk stratification and preventive strategies.

## 2 Methods

### 2.1 Study design and population

This study utilized data from a retrospective cohort of participants derived from the NHANES 2003–2018 database. The cohort was established by the National Center for Health Statistics (NCHS) using a complex multistage probability sampling design to represent non-institutionalized U.S. residents. Our study initially included 80,312 participants from the NHANES 2003–2018 cycles.

**Fig. 1:**
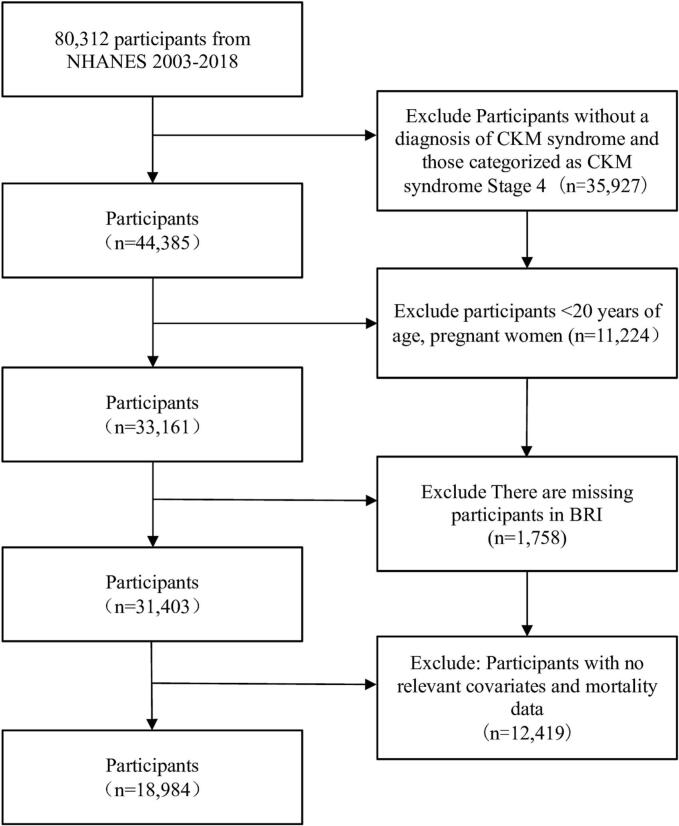
Participants screening flowchart.

The exclusion criteria were as follows: (1) participants without a diagnosis of CKM syndrome and those with stage 4 CKM syndrome (*n* = 35,927); (2) age < 20 years and pregnant women (*n* = 11,224); (3) missing BRI values (*n* = 1758); (4) lack of relevant covariates and mortality data (*n* = 12,419). The participant screening flowchart is shown in Fig. 1. Through this process, 18,984 participants were included in the final analysis. The study protocol was approved by the NCHS Research Ethics Review Board, and all participants provided written informed consent. As this study utilized only de-identified, publicly available data from NHANES, no additional ethical approval was required.

### 2.2 Definitions of CKM syndrome

According to the 2023 AHA Presidential Advisory on CKM Health [5], CKM syndrome is categorized into Stages 0 through 4: Stage 0: Comprises individuals with no risk factors for CKM syndrome. Stage 1: Comprises individuals with overweight/obesity or prediabetes. Stage 2: Comprises individuals with moderate- to high-risk CKD and metabolic risk factors (including hypertension, diabetes, fasting serum triglycerides >135 mg/dL, or metabolic syndrome). Stage 3: Comprises individuals with subclinical CVD. Subclinical CVD is defined as having a predicted 10-year CVD risk ≥20% (high risk) or CKD classified as very high-risk. Stage 4: Comprises individuals with clinical CVD. CKD classification is determined using KDIGO (Kidney Disease Improving Global Outcomes) criteria, which integrate the estimated glomerular filtration rate (eGFR) and the urine albumin-to-creatinine ratio (UACR) [18]. eGFR is calculated using the 2021 race-free Chronic Kidney Disease Epidemiology Collaboration (CKD-EPI) creatinine Eq. [19]. The predicted 10-year CVD risk is calculated using the basic Predicting Risk of CVD EVENTs (PREVENT) Eq. [20]. Stage 4 participants (with clinical CVD) were excluded due to their distinct pathophysiology and higher competing risks, which could confound the association between adiposity and mortality in earlier stages (0–3). Thus, our findings apply primarily to populations without established CVD and should not be generalized to Stage 4. More detailed staging criteria are provided in Supplementary Material, Table S1.

### 2.3 Definition of BRI

The height (cm) and WC (cm) used in the BRI were obtained from the laboratory body measures dataset. To ensure data accuracy, all measurements were collected by trained health technicians. The calculation formula [10] is 364.2−365.5×√1−WCcm/2π^2/0.5×Heightcm^2. Participants were divided into 5 groups according to the 20th, 40th, 60th, and 80th quintiles to investigate the relationship with all-cause mortality and cardiovascular mortality risk in stages 0–3 of CKM syndrome.

### 2.4 Ascertainment of outcomes

The mortality data used in this study were obtained from the NHANES Public-use Linked Mortality File. This file was created by linking the cohort database with the National Death Index (NDI). All-cause mortality was defined as death from any cause, while cardiovascular mortality was defined as death specifically caused by heart disease. The underlying cause of death and multiple causes of death were classified according to the International Classification of Diseases, Tenth Revision (ICD-10). The follow-up period extended from the date of the initial interview until either the date of death or December 31, 2019, whichever occurred first.

### 2.5 Covariates

Comprehensive data were collected from participants, including demographic characteristics, physical examination results, laboratory test findings, lifestyle habits, and medical history. Demographic information including age, gender, ethnicity, education, and poverty-income ratio (PIR) was assessed as self-reported variables based on standardized interview classifications. Physical examination measurements consisted of BMI, height, weight, and WC. Lifestyle factors encompassed smoking (categorized as never, former, or current), alcohol consumption (classified as current, former, or non-drinker), physical activity, and daily energy intake. Alcohol consumption was classified based on self-reported intake: “current drinkers” were defined as those who consumed at least 12 alcoholic drinks in the past year; “former drinkers” as those who reported past consumption but none in the past year; and “non-drinkers” as those who never consumed alcohol. Physical activity was evaluated according to the U.S. Physical Activity Guidelines, which recommend that adults engage in at least 150 min of moderate-intensity exercise, 75 min of vigorous-intensity exercise, or an equivalent combination of both per week [21].

Medical history data were collected according to standardized diagnostic criteria. Hypertension was defined based on self-reported physician diagnosis, current use of antihypertensive medications, or an average blood pressure measurement ≥140/90 mmHg [22]. Hyperlipidemia was diagnosed when meeting any of the following criteria: triglycerides (TG) ≥200 mg/dL, total cholesterol (TC) ≥240 mg/dL, low-density lipoprotein cholesterol (LDL-C) ≥160 mg/dL, high-density lipoprotein cholesterol (HDL—C) <40 mg/dL (men) or < 50 mg/dL (women), or current use of lipid-lowering medications [23]. Diabetes mellitus was defined by self-reported physician diagnosis, use of oral hypoglycemic agents or insulin, fasting blood glucose level > 126 mg/dL, oral glucose tolerance test (OGTT) ≥11.1 mmol/L, or hemoglobin A1c (HbA1c) ≥6.5% [24]. Metabolic syndrome was identified by the presence of three or more of the following components: WC ≥ 102 cm in men or ≥ 88 cm in women; HDL-C < 40 mg/dL in men or < 50 mg/dL in women; TG ≥150 mg/dL; elevated blood pressure (≥130/80 mmHg, medical diagnosis, or use of antihypertensive medications); and fasting blood glucose (FBG) ≥100 mg/dL [23]. CKD was classified into low, moderate, high, and very high-risk categories based on estimated glomerular filtration rate (eGFR) and urinary albumin-to-creatinine ratio (UACR) according to KDIGO guidelines [18].

Laboratory test results included: HbA1c, total cholesterol (TC), high-density lipoprotein cholesterol (HDL—C), estimated glomerular filtration rate (eGFR), serum creatinine (Scr), and urinary albumin-to-creatinine ratio (UACR).

### 2.6 Statistical analysis

To account for the complex, multistage sampling design of NHANES, all analyses incorporated survey design elements, including sample weights, clustering, and stratification. Kolmogorov-Smirnov (K—S) tests were performed to assess the normality of continuous variables. Continuous variables are presented as weighted median and interquartile range (IQR), and categorical variables as weighted percentage with 95% confidence interval (CI). Weighted one-way analysis of variance (ANOVA) was used for continuous variables, and weighted chi-square tests were employed for categorical variables.

Cox proportional hazards regression models were employed to analyze the association between BRI and the risks of all-cause and cardiovascular mortality in patients with stages 0–3 CKM syndrome. To account for competing risks (e.g., non-cardiovascular deaths), Fine-Gray proportional subdistribution hazards models were additionally used for cardiovascular mortality. Results are presented as hazard ratios (HR) and subdistribution hazard ratios (sHR) with corresponding 95% CI, respectively. Additionally, linear trends across BRI quintiles were tested by modeling the median value within each quintile as a continuous variable. Three models were constructed: Model 1 was unadjusted; Model 2 was adjusted for age, gender, ethnicity, education, PIR; and Model 3 was further adjusted for age, gender, ethnicity, education, PIR, smoking, alcohol consumption, hypertension, hyperlipidemia, daily energy intake, and physical activity.

To evaluate potential nonlinear dose-response relationships between BRI and the risks of all-cause and cardiovascular mortality in patients with stages 0–3 CKM syndrome, restricted cubic splines (RCS) were applied. If the RCS analysis indicated a nonlinear association, threshold effect analysis was further conducted to precisely identify and quantify any significant inflection points at which the relationship between BRI and mortality risks substantively changed. Subgroup analyses were performed to examine the association between BRI and the risks of all-cause and cardiovascular mortality in patients with stages 0–3 CKM syndrome, stratified by gender, age, ethnicity, education, PIR, smoking, alcohol consumption, hypertension, and hyperlipidemia. To address multiple comparisons in subgroup analyses, effect modification was assessed by testing the statistical significance of interaction terms between BRI and each stratification variable, rather than by comparing separate subgroup estimates. Statistical significance for interaction was determined using the likelihood ratio test (LRT) on the coefficient of the interaction term. Finally, to evaluate the robustness of the association results, sensitivity analyses were conducted by excluding participants who died within two years after enrollment. All analyses were performed using R software (version 4.4.2). Two-sided *p*-values <0.05 were considered statistically significant.

## 3 Results

### 3.1 Baseline characteristics

This study included 18,984 participants. During a median follow-up period of 7.5 years, 1756 of participants died from all causes, and 471 died from CVD. The baseline characteristics stratified by BRI quintiles are presented in Table 1. Significant differences (*P* < 0.05) were observed across all BRI quintiles for every baseline characteristic, including age, gender, ethnicity, education, PIR, BMI, WC, height, weight, TC, HDL—C, HbA1c, UACR, Scr, eGFR, daily energy intake, smoking, alcohol consumption, physical activity, hypertension, hyperlipidemia, diabetes, MetS, and CKD. Compared to individuals in the middle BRI quintile (Q3), participants in the highest BRI quintile (Q5) were more likely to be female, self-identify as Mexican American or non-Hispanic Black, have a high school education or below, exhibit a lower PIR, and report a physically inactive lifestyle. Clinically, they exhibited significantly higher BMI, weight, WC, TC, HbA1c, UACR, and Scr, along with a higher prevalence of hypertension, hyperlipidemia, diabetes, MetS, and CKD. Meanwhile, their eGFR levels were also notably elevated.

**Table 1:** Baseline characteristics of the study population based on BRI quintiles.^a^, ^b^, ^c^

Characteristics	BRI (quintiles)						*P*-value
	Q1 [1.21, 3.93]	Q2 (3.93, 4.88]	Q3 (4.88, 5.85]	Q4 (5.85, 7.27]	Q5 (7.27, 20.5]	Overall	
n	3798	3796	3796	3798	3796	18,984	
Age, years, median (IQR)	38.00 (27.00, 52.00)	47.00 (35.00, 57.00)	50.00 (38.00, 61.00)	52.00 (39.00, 63.00)	50.00 (36.00, 61.00)	47.00 (34.00, 59.00)	<0.001
Gender, % (95%CI)							<0.001
Female	50.28 (48.05, 52.50)	44.32 (41.59, 47.06)	46.63 (44.57, 48.70)	49.26 (47.03, 51.49)	64.48 (62.24, 66.71)	50.68 (49.68, 51.68)	
Male	49.72 (47.50, 51.95)	55.68 (52.94, 58.41)	53.37 (51.30, 55.43)	50.74 (48.51, 52.97)	35.52 (33.29, 37.76)	49.32 (48.32, 50.32)	
Ethnicity, % (95%CI)							<0.001
Mexican American	4.17 (3.17, 5.18)	7.69 (6.11, 9.26)	9.66 (7.84, 11.48)	10.71 (8.59, 12.82)	11.02 (8.65, 13.40)	8.50 (6.96, 10.04)	
Non-Hispanic Asian	6.46 (5.06, 7.86)	4.29 (3.33, 5.25)	2.80 (2.15, 3.46)	1.45 (1.08, 1.82)	0.86 (0.59, 1.13)	3.30 (2.68, 3.91)	
Non-Hispanic Black	10.64 (9.26, 12.01)	8.39 (6.97, 9.80)	8.49 (7.14, 9.84)	9.53 (7.97, 11.10)	13.07 (10.80, 15.33)	9.96 (8.63, 11.30)	
Non-Hispanic White	70.20 (67.45, 72.96)	70.96 (67.88, 74.04)	70.18 (67.05, 73.30)	69.25 (65.90, 72.60)	66.54 (62.77, 70.30)	69.51 (66.81, 72.20)	
Other Hispanic	4.10 (3.17, 5.04)	5.07 (3.96, 6.18)	5.75 (4.64, 6.86)	5.79 (4.60, 6.98)	5.04 (4.08, 6.00)	5.13 (4.27, 5.99)	
Other Race	4.43 (3.38, 5.48)	3.61 (2.80, 4.42)	3.12 (2.37, 3.87)	3.27 (2.41, 4.14)	3.47 (2.43, 4.51)	3.60 (3.16, 4.03)	
Education, % (95%CI)							<0.001
<High school diploma	10.03 (8.68, 11.38)	11.63 (10.15, 13.10)	15.02 (13.22, 16.81)	16.93 (15.04, 18.81)	16.76 (14.91, 18.61)	13.90 (12.72, 15.07)	
≥College degree	69.77 (66.87, 72.67)	66.09 (63.30, 68.88)	62.58 (59.67, 65.48)	58.21 (55.52, 60.91)	57.51 (54.89, 60.13)	63.13 (61.28, 64.97)	
High school graduate	20.20 (17.95, 22.45)	22.28 (19.98, 24.59)	22.41 (20.31, 24.51)	24.86 (22.48, 27.24)	25.73 (23.36, 28.11)	22.98 (21.72, 24.23)	
PIR, % (95%CI)							<0.001
<2.3	35.58 (32.78, 38.39)	33.39 (30.57, 36.21)	35.35 (32.66, 38.04)	39.28 (36.65, 41.91)	45.55 (42.51, 48.59)	37.59 (35.67, 39.51)	
≥2.3	64.42 (61.61, 67.22)	66.61 (63.79, 69.43)	64.65 (61.96, 67.34)	60.72 (58.09, 63.35)	54.45 (51.41, 57.49)	62.41 (60.49, 64.33)	
BMI, kg/m^2^, median (IQR)	23.14 (21.24, 25.22)	26.59 (25.25, 28.12)	29.02 (27.37, 30.80)	31.97 (30.22, 33.90)	38.07 (35.28, 42.20)	28.72 (25.53, 32.96)	<0.001
WC, cm, median (IQR)	83.00 (78.00, 88.50)	94.20 (90.00, 98.30)	101.00 (96.30, 105.70)	108.00 (103.40, 112.90)	121.40 (114.60, 129.80)	100.00 (90.50, 110.20)	<0.001
Height, cm, median (IQR)	170.50 (163.20, 177.90)	170.30 (163.20, 177.50)	169.40 (162.20, 176.90)	167.70 (161.00, 175.30)	165.50 (158.80, 172.90)	168.70 (161.80, 176.40)	<0.001
Weight, kg, median (IQR)	67.10 (58.40, 76.80)	77.60 (68.40, 87.00)	83.40 (74.30, 93.40)	90.90 (80.40, 101.80)	106.30 (92.90, 122.00)	82.80 (70.60, 96.80)	<0.001
TC, mg/dL, median (IQR)	186.00 (160.00, 215.00)	199.00 (174.00, 228.00)	201.00 (176.00, 231.00)	198.00 (172.00, 226.00)	194.00 (169.00, 222.00)	196.00 (170.00, 224.00)	<0.001
HDL-C, mg/Dl, median (IQR)	57.00 (46.00, 70.00)	51.00 (42.00, 62.00)	49.00 (40.00, 60.00)	46.00 (39.00, 56.00)	46.00 (39.00, 54.00)	49.00 (41.00, 61.00)	<0.001
HbA1c, %, median (IQR)	5.30 (5.00, 5.50)	5.40 (5.10, 5.60)	5.50 (5.20, 5.70)	5.50 (5.30, 5.90)	5.70 (5.40, 6.10)	5.40 (5.20, 5.70)	<0.001
UACR, mg/g, mean (SD)	5.84 (4.04, 10.42)	5.62 (3.87, 9.21)	6.23 (4.21, 10.36)	6.64 (4.47, 11.99)	7.67 (4.85, 15.19)	6.29 (4.21, 11.11)	<0.001
Scr, mg/dL, median (IQR)	0.85 (0.72, 1.00)	0.88 (0.74, 1.00)	0.87 (0.73, 1.01)	0.86 (0.72, 1.00)	0.80 (0.70, 0.93)	0.85 (0.72, 1.00)	<0.001
eGFR, mL/min/1.73 m^2^, median (IQR))	102.03 (88.33, 113.66)	97.89 (84.65, 109.31)	96.06 (81.35, 108.11)	95.74 (81.47, 108.03)	98.80 (83.41, 111.36)	98.18 (84.05, 110.37)	<0.001
Daily energy intake, kcal, median (IQR)	2103.00 (1577.00, 2824.00)	2037.00 (1537.00, 2648.00)	2020.00 (1521.00, 2635.00)	1988.00 (1480.00, 2630.00)	1981.00 (1476.00, 2569.00)	2032.00 (1518.00, 2669.00)	<0.001
Smoking, % (95%CI)							<0.001
Current	23.69 (21.32, 26.06)	20.04 (18.02, 22.06)	18.42 (16.80, 20.05)	17.90 (16.34, 19.46)	16.98 (15.46, 18.49)	19.54 (18.45, 20.63)	
Former	19.87 (17.97, 21.77)	24.13 (22.15, 26.12)	27.11 (24.77, 29.45)	29.68 (27.53, 31.84)	27.44 (25.19, 29.69)	25.46 (24.43, 26.50)	
Never	56.44 (53.66, 59.22)	55.82 (53.16, 58.49)	54.47 (52.10, 56.84)	52.42 (50.29, 54.54)	55.58 (53.19, 57.97)	55.00 (53.65, 56.34)	
Alcohol consumption, % (95%CI)							<0.001
Current Drinker	83.81 (81.90, 85.72)	80.55 (78.32, 82.79)	79.71 (77.71, 81.72)	76.29 (74.01, 78.56)	71.72 (69.30, 74.15)	78.67 (77.21, 80.14)	
Former Drinker	8.10 (7.03, 9.17)	9.36 (8.07, 10.65)	9.59 (8.25, 10.92)	11.54 (10.05, 13.03)	15.07 (13.18, 16.96)	10.59 (9.78, 11.40)	
Non-Drinker	8.09 (6.58, 9.59)	10.09 (8.34, 11.84)	10.70 (9.30, 12.10)	12.17 (10.53, 13.81)	13.21 (11.64, 14.77)	10.74 (9.66, 11.82)	
Physical Activity, % (95%CI)							<0.001
Active	75.02 (73.33, 76.71)	68.61 (66.16, 71.07)	63.37 (61.29, 65.45)	59.65 (57.55, 61.76)	51.71 (49.14, 54.29)	64.17 (63.04, 65.31)	
Inactive	24.98 (23.29, 26.67)	31.39 (28.93, 33.84)	36.63 (34.55, 38.71)	40.35 (38.24, 42.45)	48.29 (45.71, 50.86)	35.83 (34.69, 36.96)	
Hypertension, % (95%CI)							<0.001
No	65.38 (63.31, 67.45)	52.40 (50.16, 54.64)	44.64 (42.38, 46.90)	38.17 (36.07, 40.27)	33.07 (30.98, 35.16)	47.44 (46.25, 48.62)	
Yes	34.62 (32.55, 36.69)	47.60 (45.36, 49.84)	55.36 (53.10, 57.62)	61.83 (59.73, 63.93)	66.93 (64.84, 69.02)	52.56 (51.38, 53.75)	
Hyperlipemia, % (95%CI)							<0.001
No	54.13 (51.50, 56.76)	37.61 (35.37, 39.85)	30.12 (28.07, 32.18)	25.80 (23.76, 27.84)	23.63 (21.53, 25.73)	34.90 (33.69, 36.11)	
Yes	45.87 (43.24, 48.50)	62.39 (60.15, 64.63)	69.88 (67.82, 71.93)	74.20 (72.16, 76.24)	76.37 (74.27, 78.47)	65.10 (63.89, 66.31)	
Diabetes, % (95%CI)							<0.001
No	95.47 (94.53, 96.41)	93.29 (92.19, 94.39)	88.48 (87.09, 89.87)	81.67 (79.82, 83.51)	72.54 (70.61, 74.46)	86.81 (86.09, 87.54)	
Yes	4.53 (3.59, 5.47)	6.71 (5.61, 7.81)	11.52 (10.13, 12.91)	18.33 (16.49, 20.18)	27.46 (25.54, 29.39)	13.19 (12.46, 13.91)	
Mets, % (95%CI)							<0.001
No	97.87 (97.18, 98.55)	89.41 (88.01, 90.81)	76.01 (74.28, 77.74)	66.38 (64.47, 68.28)	56.30 (54.14, 58.45)	78.16 (77.36, 78.96)	
Yes	2.13 (1.45, 2.82)	10.59 (9.19, 11.99)	23.99 (22.26, 25.72)	33.62 (31.72, 35.53)	43.70 (41.55, 45.86)	21.84 (21.04, 22.64)	
CKD, % (95%CI)							<0.001
High	1.22 (0.78, 1.67)	0.97 (0.59, 1.34)	1.39 (1.02, 1.77)	2.84 (2.22, 3.47)	3.56 (2.78, 4.34)	1.94 (1.70, 2.17)	
Low	89.20 (87.84, 90.55)	91.42 (90.23, 92.62)	89.66 (88.34, 90.97)	85.36 (83.87, 86.86)	82.14 (80.43, 83.85)	87.75 (87.11, 88.38)	
Moderately high	9.30 (7.97, 10.62)	7.28 (6.19, 8.37)	8.34 (7.12, 9.55)	11.16 (9.81, 12.50)	13.03 (11.58, 14.49)	9.71 (9.14, 10.28)	
Very high	0.28 (0.16, 0.41)	0.33 (0.16, 0.49)	0.61 (0.38, 0.84)	0.64 (0.40, 0.87)	1.26 (0.89, 1.64)	0.60 (0.50, 0.71)	

a BRI: Body roundness index, PIR: Poverty-to-income ratio, BMI: Body mass index, WC: Waist circumference, TC: Total cholesterol, HDL-C: High-density lipoprotein cholesterol, HbA1c: glycosylated hemoglobin, UACR: Urine albumin creatine ratio, Scr: Serum creatinine, eGFR: estimated glomerular filtration rate, Mets: Metabolic Syndrome, CKD: Chronic kidney disease.

b Continuous variables are presented as weighted median and interquartile range (IQR), and categorical variables as weighted percentage with 95% confidence interval (CI).

c * *P*-values were obtained by one-way ANOVA or chi-square test, and a *P*-value of <0.05 was considered significant.

### 3.2 Association of BRI with all-cause and cardiovascular mortality

Cox proportional hazards regression analysis revealed a significant positive association between higher BRI values and increased risk of all-cause mortality (Table 2). In the unadjusted model (Model 1), each unit increase in BRI was associated with a 10% elevated risk of all-cause mortality (HR = 1.10, 95% *CI*: 1.07–1.13, *P*-value <0.001). Participants in the highest BRI quintile (Q5) had 1.36 times the risk of all-cause mortality compared with those in the middle quintile (Q3) (HR = 1.36, 95% *CI*: 1.08–1.71, *P*-value = 0.009). After full adjustment for demographic, lifestyle, dietary, and clinical covariates (Model 3), each unit increase in BRI remained significantly associated with a 4% increase in risk (HR = 1.04, 95% *CI*: 1.01–1.08, *P*-value = 0.025). Similarly, individuals in the highest quintile (Q5) continued to exhibit a 34% higher risk than those in the middle quintile (Q3) (HR = 1.34, 95% *CI*: 1.08–1.66, *P*-value = 0.008). No significant linear trend was observed in Model 3 (*P* for trend >0.05), suggesting the possible presence of a nonlinear association.

**Table 2:** Association of BRI with all-cause mortality and cardiovascular mortality.^a^, ^b^, ^c^, ⁎

	HR (95% *CI*), *P*-value		
	Model 1	Model 2	Model 3
All-cause mortality			
BRI	1.10(1.07, 1.13), <0.001	1.05(1.01, 1.09), 0.008	1.04(1.01, 1.08), 0.025
BRI (quintiles)			
Q1	0.70(0.57, 0.85), <0.001	1.26(0.99, 1.59), 0.061	1.24(0.97, 1.59), 0.091
Q2	0.88(0.71, 1.08), 0.211	1.07(0.87, 1.32), 0.520	1.05(0.85, 1.28), 0.667
Q3	1	1	1
Q4	1.33(1.08, 1.65), 0.007	1.20(0.98, 1.46), 0.076	1,15(0.93, 1.42), 0.188
Q5	1.36(1.08, 1.71), 0.009	1.42(1.15, 1.75), 0.001	1.34(1.08, 1.66), 0.008
*P*-trend	<0.001	0.034	0.099
Cardiovascular mortality			
BRI	1.13(1.07, 1.20), <0.001	1.13(1.07, 1.20), <0.001	1.13(1.06, 1.20), <0.001
BRI (quintiles)			
Q1	1.01(0.65, 1.56), 0.979	1.07(0.72, 1.60), 0.728	1.09(0.75, 1.59), 0.649
Q2	1.04(0.66, 1.63), 0.877	1.15(0.75, 1.75), 0.531	1.15(0.75, 1.76), 0.518
Q3	1	1	1
Q4	1.37(0.91, 2.08), 0.136	1.33(0.89, 1.99), 0.167	1,33(0.89, 1.98), 0.164
Q5	1.96(1.33, 2.87), <0.001	2.07(1.42, 3.01), <0.001	2.01(1.38, 2.93), <0.001
*P*-trend	<0.001	<0.001	<0.001

a Model 1 was unadjusted.

b Model 2 was adjusted for age, gender, ethnicity, education, PIR.

c Model 3 was adjusted for age, gender, ethnicity, education, PIR, smoking, alcohol consumption, hypertension, hyperlipidemia, daily energy intake, and physical activity.

⁎ BRI: Body roundness index, HR: Hazard ratio, 95%CI: 95% Confidence intervals.

For cardiovascular mortality, a significant positive association was observed between higher BRI values and increased risk of cardiovascular mortality, with competing risks from non-cardiovascular deaths accounted for using Fine-Gray proportional subdistribution hazards models (Table 3). In the unadjusted model (Model 1), each unit increase in BRI was associated with a 10% elevated risk of cardiovascular mortality (SHR = 1.10, 95% *CI*: 1.06–1.14, *P* < 0.001). Participants in the highest BRI quintile (Q5) had a 54% higher risk of cardiovascular mortality compared to those in the middle quintile (Q3) (SHR = 1.54, 95% *CI*: 1.17–2.03, *P* = 0.0023). After full adjustment for demographic, lifestyle, dietary, and clinical covariates (Model 3), each unit increase in BRI remained significantly associated with an 8% increase in risk (SHR = 1.08, 95% *CI*: 1.03–1.13, *P* = 0.001). Similarly, individuals in the highest quintile (Q5) continued to exhibit a 61% higher risk than those in the middle quintile (Q3) (SHR = 1.61, 95% *CI*: 1.21–2.13, *P* < 0.001). A significant linear trend was observed in Model 3 (*P* for trend = 0.008). For comparison, the results from the standard Cox proportional hazards models (which do not account for competing risks) were consistent in direction and magnitude (Table 2), with each unit increase in BRI associated with a 13% higher risk (HR = 1.13, 95% *CI*: 1.06–1.20, *P*-value <0.001) and the highest BRI quintile showing approximately twice the risk (HR = 2.01, 95% *CI*: 1.38–2.93, *P*-value <0.001) after full adjustment, also showing a significant linear trend (*P* for trend <0.001).

**Table 3:** Association of BRI with all-cause mortality and cardiovascular mortality using Fine-Gray proportional subdistribution hazards models.^a^, ^b^, ^c^, ⁎

	SHR (95% CI), *P*-value		
	Model 1	Model 2	Model 3
Cardiovascular mortality			
BRI	1.10(1.06, 1.14), <0.001	1.08(1.03, 1.13), 0.001	1.08(1.03, 1.13), 0.001
BRI (quintiles)			
Q1	0.80(0.58, 1.10), <0.001	1.27(0.91, 1.76), 0.154	1.21(0.87, 1.68), 0.261
Q2	0.97(0.71, 1.31), 0.825	1.13(0.84, 1.53), 0.424	1.11(0.82, 1.51), 0.484
Q3	1	1	1
Q4	1.52(1.15, 2.00), 0.0030	1.32(1.00, 173), 0.051	1,30(0.98, 1.71), 0.065
Q5	1.54(1.17, 2.03), 0.0023	1.67(1.26, 2.21), <0.001	1.61(1.21, 2.13), <0.001
*P*-trend	<0.001	0.006	0.008

a Model 1 was unadjusted.

b Model 2 was adjusted for age, gender, ethnicity, education, PIR.

c Model 3 was adjusted for age, gender, ethnicity, education, PIR, smoking, alcohol consumption, hypertension, hyperlipidemia, daily energy intake, and physical activity.

⁎ BRI: Body roundness index, SHR: subdistribution Hazard ratio, 95%CI: 95% Confidence intervals.

### 3.3 Restricted cubic spline analyses

**Fig. 2:**
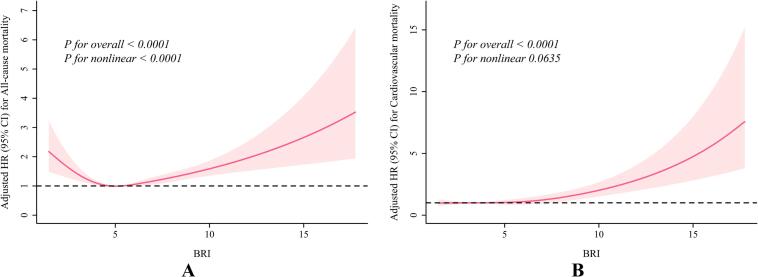
The association of body roundness index with all-cause and cardiovascular mortality in patients with CKM syndrome stage 0–3 visualized by restricted cubic spline. Sample analyses were weighted and model 3 was adjusted for age, gender, ethnicity, education, PIR, smoking, alcohol consumption, hypertension, hyperlipidemia, daily energy intake, physical activity based on the cox proportional hazards models.

We employed RCS to evaluate the dose-response relationship between BRI and the risks of all-cause and cardiovascular mortality in patients with stages 0–3 CKM syndrome. The results revealed a significant U-shaped nonlinear association between BRI and all-cause mortality risk (*P* for overall <0.0001, *P* for nonlinear <0.0001; Fig. 2A). The risk reached its nadir at a BRI of approximately 5.5, indicating that both lower and higher values were associated with increased risk. In contrast, a significant linear association was observed between BRI and cardiovascular mortality risk (*P* for overall <0.0001, *P* for nonlinear = 0.0635; Fig. 2B). Cardiovascular mortality risk increased progressively across the BRI range. The U-shaped curve for all-cause mortality underscores the potential hazards of both underweight (potentially reflecting malnutrition or frailty) and obesity, whereas the linear relationship for cardiovascular mortality reinforces BRI as a continuous risk marker for CVD-specific outcomes in this population.

### 3.4 Threshold effect analysis

Threshold analysis confirmed the nonlinear relationship between BRI and all-cause mortality risk in patients with stages 0–3 CKM syndrome, identifying an inflection point at BRI = 5.68 (Table 4). Above this threshold (BRI ≥ 5.68), each 1-unit increase in BRI was associated with a 12% elevated risk of all-cause mortality (HR = 1.12, 95% *CI*: 1.08–1.17, *P*-value <0.0001). In contrast, below this threshold (BRI < 5.68), higher BRI values were significantly associated with a reduced risk (HR = 0.90, 95% *CI*: 0.83–0.96, *P*-value = 0.0024). The significance of this threshold effect was further confirmed by the likelihood ratio test (LRT, *P*-value <0.0001).

**Table 4:** Analysis of the threshold effect between BRI and all-cause mortality.

BRI	Adjusted HR (95%CI), P-value
Inflection point	5.68
<5.68	0.90(0.83–0.96), 0.0024
≥5.68	1.12(1.08–1.17), <0.0001
Log-likelihood ratio test	<0.0001

*Two-piecewise cox proportional hazards model was used to calculate the threshold effect of the BRI. If the log likelihood ratio test <0.05, it means the two-piecewise cox proportional hazards model is superior to the single-line cox proportional hazards model. * Model was adjusted for age, gender, ethnicity, education, PIR, smoking, alcohol consumption, hypertension, hyperlipidemia, daily energy intake, and physical activity. *BRI: Body roundness index, HR: Hazard ratio, 95%CI: 95% Confidence intervals.

### 3.5 Subgroup analyses

To evaluate potential effect modification, subgroup analyses were conducted across demographic, socioeconomic, and clinical variables, including age, gender, ethnicity, education, PIR, smoking, alcohol consumption, hypertension, and hyperlipidemia. As shown in Table 5, no significant interactions were found for age, gender, ethnicity, PIR, smoking, hypertension, or hyperlipidemia (all P for interaction >0.05). However, statistically significant interactions were identified for education level (P for interaction = 0.039) and alcohol consumption (P for interaction = 0.048). Specifically, a significant U-shaped association was observed for education level; the association between BRI and all-cause mortality was strongest among participants with less than a high school diploma, where both the lowest (Q1) and highest (Q5) BRI quintiles had significantly elevated mortality risk (HR = 1.87, 95% CI: 1.29–2.69 and HR = 1.42, 95% CI: 1.07–1.90, respectively). Similarly, among current drinkers, higher BRI was associated with a graded increase in mortality risk, with the highest quintile (Q5) showing a 36% higher risk (HR = 1.36, 95% CI: 1.00–1.85). As shown in Table 6, no significant interactions were found for gender, ethnicity, education, PIR, alcohol consumption, hypertension, or hyperlipidemia (all *P* for interaction >0.05). However, statistically significant interactions were identified for age and smoking (*P* for interaction <0.05). Specifically, among individuals under 50 years of age, high BRI was significantly associated with higher cardiovascular mortality risk (HR = 1.41, 95% *CI*: 1.18–1.68, *P*-value <0.001), while among current smokers, higher BRI was associated with a significantly elevated cardiovascular mortality risk (HR = 1.29, 95% *CI*: 1.14–1.46, *P*-value <0.001).

**Table 5:** Subgroup analysis for the association of BRI quintiles with all-cause mortality.⁎

Subgroup	BRI (quintiles), HR (95% *CI*)					*P* for interaction
	Q1 [1.21, 3.93]	Q2 (3.93, 4.88]	Q3 (4.88, 5.85]	Q4 (5.85, 7.27]	Q5 (7.27, 20.5]	
Gender						0.361
Female	1.06 (0.72–1.57)	1.01 (0.70–1.46)	1.00 (ref)	0.93 (0.67–1.29)	0.97 (0.71–1.33)	
Male	1.29 (0.95–1.76)	1.09 (0.78–1.53)	1.00 (ref)	1.34 (0.99–1.82)	1.53 (1.07–2.20)	
Age						0.258
<50	0.71 (0.37–1.39)	1.02 (0.56–1.85)	1.00 (ref)	0.94 (0.49–1.82)	1.43 (0.89–2.28)	
≥50	1.34 (1.06–1.71)	1.06 (0.86–1.30)	1.00 (ref)	1.18 (0.96–1.46)	1.20 (0.95–1.51)	
Ethnicity						0.271
Other Race	1.42 (1.02–1.98)	0.93 (0.67–1.29)	1.00 (ref)	1.10 (0.76–1.58)	1.45 (1.06–2.00)	
White	1.12 (0.84–1.51)	1.08 (0.85–1.38)	1.00 (ref)	1.16 (0.91–1.47)	1.18 (0.89–1.56)	
Education						**0.039**
<High school diploma	1.87 (1.29–2.69)	1.24 (0.87–1.78)	1.00 (ref)	1.18 (0.83–1.69)	1.42 (1.07–1.90)	
≥ College degree	0.85 (0.60–1.21)	1.07 (0.80–1.41)	1.00 (ref)	1.24 (0.90–1.70)	1.19 (0.81–1.76)	
High school graduate	1.60 (1.02–2.51)	0.99 (0.65–1.50)	1.00 (ref)	1.01 (0.68–1.51)	1.22 (0.85–1.75)	
PIR						0.306
<2.3	1.09 (0.78–1.53)	0.93 (0.69–1.25)	1.00 (ref)	1.14 (0.91–1.44)	1.03 (0.78–1.37)	
≥2.3	1.26 (0.90–1.75)	1.17 (0.84–1.61)	1.00 (ref)	1.10 (0.76–1.59)	1.49 (1.02–2.16)	
Smoking						0.415
Current	1.45 (0.94–2.24)	1.27 (0.79–2.04)	1.00 (ref)	1.26 (0.73–2.19)	1.85 (1.14–3.00)	
Former	1.16 (0.79–1.70)	0.94 (0.67–1.34)	1.00 (ref)	1.00 (0.80–1.26)	1.09 (0.80–1.50)	
Never	1.07 (0.77–1.49)	1.09 (0.81–1.47)	1.00 (ref)	1.26 (0.93–1.72)	1.14 (0.84–1.54)	
Alcohol consumption						**0.048**
Current	1.00 (0.75–1.34)	1.04 (0.78–1.38)	1.00 (ref)	1.12 (0.84–1.48)	1.36 (1.00–1.85)	
Former	2.26 (1.07–4.78)	1.09 (0.56–2.11)	1.00 (ref)	1.64 (1.00–2.67)	1.36 (0.77–2.39)	
Never	1.36 (0.78–2.38)	1.26 (0.77–2.05)	1.00 (ref)	0.95 (0.57–1.58)	0.77 (0.48–1.26)	
Hypertension						0.392
Yes	1.19 (0.92–1.52)	1.06 (0.83–1.35)	1.00 (ref)	1.05 (0.84–1.31)	1.22 (0.97–1.53)	
No	1.21 (0.68–2.15)	1.05 (0.63–1.74)	1.00 (ref)	1.59 (0.92–2.74)	1.20 (0.70–2.05)	
Hyperlipidemia						0.777
Yes	1.31 (1.00–1.71)	1.09 (0.87–1.38)	1.00 (ref)	1.16 (0.89–1.51)	1.27 (0.98–1.65)	
No	0.98 (0.65–1.48)	0.95 (0.58–1.56)	1.00 (ref)	1.13 (0.75–1.70)	1.12 (0.68–1.83)	

This analysis was adjusted for age, gender, ethnicity, education, PIR, smoking, drinking, hypertension, hyperlipidemia, daily energy intake, physical activity and insurance.

⁎ BRI: Body roundness index, HR: Hazard ratio, 95%CI: 95% Confidence intervals, CKM syndrome: Cardiovascular-Kidney-Metabolic syndrome.

**Table 6:** Subgroup analysis for the association of BRI with cardiovascular mortality.⁎

Subgroup	HR (95% *CI*)	*P*-value	*P* for interaction
Gender			0.744
Female	1.10(1.01,1.20)	0.037	
Male	1.11(1.02,1.20)	0.016	
Age			0.002
<50	1.41(1.18,1.68)	<0.001	
≥50	1.07(1.01,1.14)	0.028	
Ethnicity			0.251
Other Race	1.08(0.96,1.22)	0.200	
White	1.12(1.04,1.20)	0.002	
Education			0.536
<High school diploma	1.14(1.04,1.25)	0.007	
≥ College degree	1.14(1.03,1.27)	0.015	
High school graduate	1.03(0.94,1.13)	0.532	
PIR			0.991
<2.3	1.10(1.03,1.18)	0.004	
≥2.3	1.11 (0.99,1.24)	0.053	
Smoking			0.004
Current	1.29(1.14,1.46)	<0.001	
Former	1.12(1.02,1.24)	0.021	
Never	1.03(0.97,1.10)	0.311	
Alcohol consumption			0.742
Current	1.13(1.06,1.22)	<0.001	
Former	1.09(0.93,1.27)	0.299	
Never	1.08(0.95,1.23)	0.253	
Hypertension			0.124
Yes	1.09(1.03,1.16)	0.003	
No	1.23(1.06,1,43)	0.007	
Hyperlipidemia			0.237
Yes	1.14(1.06,1.22)	<0.001	
No	1.09(0.99,1.20)	0.089	

This analysis was adjusted for age, gender, ethnicity, education, PIR smoking, alcohol consumption, hypertension, hyperlipidemia, daily energy intake, and physical activity.

⁎ BRI: Body roundness index, HR: Hazard ratio, 95%CI: 95% Confidence intervals, CKM syndrome: Cardiovascular-Kidney-Metabolic syndrome.

### 3.6 Sensitivity analyses

Sensitivity analyses showed that after excluding participants who died within the first two years of follow-up, the significant associations between BRI and all-cause and cardiovascular mortality persisted (Supplementary Material, Tables S2-S3).

## 4 Discussion

In this latest study involving 18,984 American patients with stages 0–3 CKM syndrome, we observed significant associations between BRI and both all-cause and cardiovascular mortality. Specifically, a U-shaped association was identified between BRI and all-cause mortality, while a linear relationship was demonstrated between BRI and cardiovascular mortality. Furthermore, threshold effect analysis revealed an inflection point for BRI at 5.68 in its association with all-cause mortality. Subgroup analyses results showed significant interactions between BRI and all-cause mortality in subgroups stratified by alcohol consumption, as well as significant interactions between BRI and cardiovascular mortality in subgroups stratified by age and smoking. In conclusion, our study indicates that BRI serves as a valuable predictive indicator for all-cause and cardiovascular mortality risk in patients with stages 0–3 CKM syndrome, potentially offering practical utility for refining early CVD prevention strategies.

Our findings demonstrate a significant positive association between BRI and both all-cause and cardiovascular mortality in individuals with stages 0–3 CKM syndrome. This positive correlation is consistent with previous research findings [25], [26]. Furthermore, this study also identified a significant nonlinear U-shaped association between BRI and all-cause mortality, while a linear correlation was observed with cardiovascular mortality. These findings align with a previous prospective NHANES-based study which similarly demonstrated a U-shaped relationship between BRI and all-cause mortality in the general population [27]. A retrospective study focusing on patients with diabetes and prediabetes demonstrated a U-shaped correlation between BRI and both all-cause and cardiovascular mortality [26]. Among U.S. adults with chronic kidney disease, BRI demonstrates a U-shaped relationship with both all-cause and cardiovascular mortality. A separate analysis of NHANES data revealed a U-shaped association between BRI and all-cause mortality as well as cardiovascular mortality in patients with chronic kidney disease [28]. This indicates that both elevated and reduced BRI levels are associated with all-cause mortality risk in individuals with stages 0–3 CKM syndrome, and maintaining these indices within an optimal range may potentially reduce risk of mortality. Conversely, a separate prospective study involving diabetic patients found a linear association between BRI and cardiovascular mortality [29]. A study on mortality risk associated with metabolic dysfunction-associated steatotic liver disease (MASLD) demonstrated a linear relationship between BRI and cardiovascular mortality [30]. Nevertheless, numerous studies have demonstrated a U-shaped nonlinear association between BRI and cardiovascular mortality. This discrepancy may reflect differences in the baseline risk profiles of the study populations. In relatively early disease stages, such as in our cohort, the detrimental metabolic effects of adipose tissue may serve as the primary driving factor. Conversely, in patients with advanced disease, lower BRI levels may reflect underlying malnutrition or a catabolic state. Consequently, the clinical implications of BRI should be interpreted within the context of the patient's specific disease stage. The precise reasons underlying these observations warrant further investigation in subsequent studies.

The linear association with cardiovascular mortality versus the U-shaped association with all-cause mortality likely reflects distinct underlying pathophysiological mechanisms. For cardiovascular mortality, the linear relationship supports a dose-dependent cumulative mechanism: BRI serves as a robust surrogate for visceral adipose tissue (VAT) [31], which functions as a metabolically active endocrine organ continuously releasing pro-inflammatory adipokines (e.g., tumor necrosis factor-α, interleukin-6, leptin) and promoting systemic inflammation, endothelial dysfunction, and atherosclerotic progression [32], [33]. As VAT accumulation increases with higher BRI, the cardiovascular system undergoes progressive hemodynamic and metabolic stress without evident threshold effects, resulting in the observed linear risk escalation [34].

Conversely, the U-shaped association between BRI and all-cause mortality reflects opposing risk factors at high and low extremes. A high BRI signifies excess visceral fat and metabolic dysfunction [35]. Conversely, a low BRI may indicate pathological states such as malnutrition, sarcopenia, or chronic wasting [36]. In CKM syndrome, a low BRI could signal poor metabolic reserves and increased susceptibility to non-cardiovascular deaths, like infections or cancer [37]. This aligns with the “obesity paradox,” where moderate adiposity may be protective in chronic illness, while low weight indicates greater vulnerability [38]. Thus, while cardiovascular mortality is primarily driven by adipose-related mechanisms, all-cause mortality is also influenced by poor nutritional and functional status at low BRI levels.

Our study revealed significant effect modifications by education level and alcohol consumption. Specifically, the association between BRI and mortality was most pronounced among participants with less than a high school diploma, exhibiting a distinct U-shaped relationship. In this subgroup, both the lowest and highest BRI quintiles were associated with significantly elevated mortality risk. The markedly higher risk linked to low BRI may reflect the influence of socioeconomic disadvantages associated with lower educational attainment, such as a higher burden of underlying chronic illness, malnutrition, or sarcopenia [35], [39]. Conversely, the risk associated with high BRI aligns with the known pathophysiology of visceral adiposity. This pattern underscores that in socioeconomically vulnerable populations, both underweight and visceral obesity confer significant risk, highlighting the complex interplay between social determinants, body composition, and health outcomes [40], [41]. Furthermore, among current alcohol consumers with stages 0–3 CKM syndrome, elevated BRI was also associated with an increased risk of all-cause mortality. This may result from the synergistic effects of alcohol and visceral adiposity. Excessive alcohol intake contributes to multi-organ damage through cardiotoxicity, hepatic steatosis, and oxidative stress [42], [43]. Concurrently, high BRI—reflecting visceral fat accumulation—amplifies systemic inflammation, insulin resistance, and dyslipidemia [7]. Their combination likely accelerates ectopic fat deposition in organs such as the liver and heart, impairing metabolic and cardiac function [44]. Additionally, among individuals under 50 years of age and current smokers, elevated BRI was associated with increased cardiovascular mortality in stages 0–3 CKM syndrome. Our findings align with previous studies underscoring the prognostic value of abdominal obesity indices in younger populations. For instance, a large cohort study found that higher levels of abdominal obesity were significantly associated with an increased risk of CVD incidence and mortality among young and middle-aged adults, independent of overall BMI [45]. Furthermore, another investigation utilizing NHANES data demonstrated that body shape indices, such as the A Body Shape Index (ABSI), were strongly predictive of cardiovascular mortality, particularly in individuals under 65 years of age, highlighting the critical role of body fat distribution beyond overall weight [46]. Among current smokers, the association between elevated BRI and increased cardiovascular mortality risk is more pronounced. Tobacco smoke activates inflammatory cells, generating elevated inflammatory cytokines (e.g., TNF-α, IL-6) and reactive oxygen species (ROS) [47]. Visceral adipose tissue—a core feature of high BRI—also secretes pro-inflammatory factors and promotes oxidative stress [48]. thereby synergistically accelerating atherosclerosis. Smoking additionally impairs endothelial function and reduces nitric oxide (NO) bioavailability, promoting thrombosis [49]. It also elevates LDL and total cholesterol while reducing HDL, further facilitating oxidized LDL deposition and foam cell formation [50]. In summary, smoking and high BRI exhibit synergistic effects through inflammation, endothelial dysfunction, and lipid metabolism, markedly amplifying cardiovascular mortality risk in smokers.

Based on these findings, BRI may serve as a practical, low-cost tool for early risk identification and stratification within the CKM spectrum. Its measurement simplicity supports potential use in primary care to flag high-risk individuals for intensified monitoring or preventive intervention. Further research is warranted to establish stage-specific BRI cutoffs and validate its utility in improving clinical outcomes.

This study has several strengths. First, we utilized the nationally representative NHANES database. Its rigorous complex survey design ensures that our findings are generalizable to the non-institutionalized adult population of the United States, enhancing the external validity of our results. Second, to our knowledge, this is the first study to thoroughly investigate the prognostic value of the BRI, a novel indicator of body fat distribution, in the context of the emerging CKM syndrome classification, providing a new perspective for early risk stratification. Third, we conducted extensive subgroup and sensitivity analyses and adjusted for a wide range of potential confounders in multivariable models. The consistency of the results robustly supports the reliability of our primary findings. However, several limitations should be acknowledged. First, due to the observational study design, although we meticulously adjusted for numerous confounders, we cannot entirely rule out the possibility of residual confounding or establish a causal relationship between BRI and mortality. Second, the staging of CKM syndrome was based on single laboratory measurements and self-reported medical history, which is susceptible to misclassification bias. Fourth, although we applied appropriate statistical models for competing risks where noted, the use of only baseline measurements for BRI and key covariates (e.g., smoking, alcohol use) means we could not account for their changes over time, which may introduce time-dependent confounding. Finally, the generalizability of our findings is limited to non-institutionalized US adults and does not extend to adolescents, individuals with established clinical cardiovascular disease (CKM Stage 4), or populations with substantially different healthcare contexts or body composition profiles.

## 5 Conclusions

In individuals with stages 0–3 CKM syndrome, we found a U-shaped association between BRI and all-cause mortality, where both low and high values conferred elevated risk, with low BRI values (e.g., <5.68) potentially indicative of underlying malnutrition or a catabolic state. A positive linear association was observed between BRI and cardiovascular mortality. These findings highlight BRI as a practical anthropometric indicator for mortality risk stratification in early-stage CKM syndrome, supporting its potential integration into routine assessment for early prevention. Future studies should investigate whether dynamic changes in BRI over time improve mortality prediction and provide further insight into its clinical utility.

## CRediT authorship contribution statement

**Zehua He:** Writing – original draft, Visualization, Formal analysis, Conceptualization. **Wenjing Xiong:** Writing – original draft, Methodology, Investigation, Data curation. **Yajing Li:** Writing – review & editing, Validation, Software, Resources. **Xia Wu:** Writing – review & editing, Visualization, Formal analysis. **Yiyun Zhang:** Writing – review & editing, Investigation, Data curation. **Xiaoyun Shan:** Writing – review & editing, Resources, Formal analysis, Data curation. **Yan Liu:** Writing – review & editing, Supervision, Project administration, Methodology. **Weiqing Rang:** Writing – review & editing, Supervision, Funding acquisition, Conceptualization.

## Consent for publication

Not applicable.

## Ethics approval and consent to participate

The National Health and Nutrition Examination Survey (NHANES) protocol was reviewed and approved by the Research Ethics Review Board of the National Center for Health Statistics (NCHS). All participants provided written informed consent prior to participation. As this study utilized only de-identified, publicly available data from NHANES, no additional ethical approval was required.

## Funding

This work was supported by the 2019 Hunan Furong Distinguished Teaching Faculty Special Fund (Grant No. 201RFS001). The funder had no role in the design of the study; in the collection, analysis, or interpretation of data; in the writing of the manuscript; or in the decision to publish the results.

## Declaration of competing interest

The authors declare no competing financial or non-financial interests that could influence the work reported in this paper.

## Data Availability

The data supporting the findings of this study are publicly available and were obtained from the National Health and Nutrition Examination Survey (NHANES), conducted by the National Center for Health Statistics (NCHS). All datasets can be accessed freely online through the NHANES website at: https://wwwn.cdc.gov/nchs/nhanes/Default.aspx.

## References

[1] Ndumele C.E., Neeland I.J., Tuttle K.R., Chow S.L., Mathew R.O., Khan S.S. (2023). A synopsis of the evidence for the science and clinical management of Cardiovascular-Kidney-Metabolic (CKM) syndrome: a scientific statement from the American Heart Association. Circulation.

[2] Aggarwal R., Ostrominski J.W., Vaduganathan M. (2024). Prevalence of cardiovascular-kidney-metabolic syndrome stages in US adults, 2011-2020. JAMA.

[3] Minhas A.M.K., Mathew R.O., Sperling L.S., Nambi V., Virani S.S., Navaneethan S.D. (2024). Prevalence of the cardiovascular-kidney-metabolic syndrome in the United States. J. Am. Coll. Cardiol..

[4] Ostrominski J.W., Arnold S.V., Butler J., Fonarow G.C., Hirsch J.S., Palli S.R. (2023). Prevalence and overlap of cardiac, renal, and metabolic conditions in US adults, 1999-2020. JAMA Cardiol..

[5] Ndumele C.E., Rangaswami J., Chow S.L., Neeland I.J., Tuttle K.R., Khan S.S. (2023). Cardiovascular-kidney-metabolic health: a presidential advisory from the American Heart Association. Circulation.

[6] Khan S.S., Coresh J., Pencina M.J., Ndumele C.E., Rangaswami J., Chow S.L. (2023). Novel prediction equations for absolute risk assessment of total cardiovascular disease incorporating cardiovascular-kidney-metabolic health: a scientific statement from the American Heart Association. Circulation.

[7] Neeland I.J., Ross R., Després J.-P., Matsuzawa Y., Yamashita S., Shai I. (2019). Visceral and ectopic fat, atherosclerosis, and cardiometabolic disease: a position statement. Lancet Diabetes Endocrinol..

[8] Roundtable on Obesity Solutions, Food and Nutrition Board, Health and Medicine Division, National Academies of Sciences, Engineering, and Medicine (2023).

[9] Shao Y., Wang N., Shao M., Liu B., Wang Y., Yang Y. (2025). The lean body mass to visceral fat mass ratio is negatively associated with cardiometabolic disorders: a cross-sectional study. Sci. Rep..

[10] Thomas D.M., Bredlau C., Bosy-Westphal A., Mueller M., Shen W., Gallagher D. (2013). Relationships between body roundness with body fat and visceral adipose tissue emerging from a new geometrical model: body roundness with body fat & visceral adipose tissue. Obesity.

[11] Qiu L., Xiao Z., Fan B., Li L., Sun G. (2024). Association of body roundness index with diabetes and prediabetes in US adults from NHANES 2007–2018: a cross-sectional study. Lipids Health Dis..

[12] Zhang J., Yu X. (2024). The association between the body roundness index and the risk of chronic kidney disease in US adults. Front. Med. (Lausanne).

[13] Rico-Martín S., Calderón-García J.F., Sánchez-Rey P., Franco-Antonio C., Martínez Alvarez M., Sánchez Muñoz-Torrero J.F. (2020). Effectiveness of body roundness index in predicting metabolic syndrome: a systematic review and meta-analysis. Obes. Rev..

[14] Yang M., Liu J., Shen Q., Chen H., Liu Y., Wang N. (2024). Body roundness index trajectories and the incidence of cardiovascular disease: evidence from the China health and retirement longitudinal study. JAHA.

[15] Xu J., Zhang L., Wu Q., Zhou Y., Jin Z., Li Z. (2021). Body roundness index is a superior indicator to associate with the cardio-metabolic risk: evidence from a cross-sectional study with 17,000 eastern-China adults. BMC Cardiovasc. Disord..

[16] Huang W., Yin H., Yang B. (2025). Investigating the relationship between body roundness index and low muscle mass based on a cross-sectional study: focus on visceral adipose tissue. PloS One.

[17] Cai J., Zheng D., Xu S., Jiang S., Jiang Y., Zhang R. (2025). Association of body roundness index with cardiovascular disease in early Cardiovascular-Kidney-Metabolic syndrome stage 0-3: mediation by the triglyceride-glucose index in a national cohort study. Diabetol. Metab. Syndr..

[18] Stevens P.E., Ahmed S.B., Carrero J.J., Foster B., Francis A., Hall R.K. (2024). KDIGO 2024 clinical practice guideline for the evaluation and management of chronic kidney disease. Kidney Int..

[19] Inker L.A., Eneanya N.D., Coresh J., Tighiouart H., Wang D., Sang Y. (2021). New creatinine- and cystatin C–based equations to estimate GFR without race. N. Engl. J. Med..

[20] Khan S.S., Matsushita K., Sang Y., Ballew S.H., Grams M.E., Surapaneni A. (2024). Development and validation of the American Heart Association’s PREVENT equations. Circulation.

[21] Piercy K.L., Troiano R.P., Ballard R.M., Carlson S.A., Fulton J.E., Galuska D.A. (2018). The physical activity guidelines for Americans. JAMA.

[22] Whelton P.K., Carey R.M., Aronow W.S., Casey D.E., Collins K.J., Dennison Himmelfarb C. (2018). 2017 ACC/AHA/AAPA/ABC/ACPM/AGS/APhA/ASH/ASPC/NMA/PCNA guideline for the prevention, detection, evaluation, and management of high blood pressure in adults. J. Am. Coll. Cardiol..

[23] Expert Panel On Detection, Evaluation, And Treatment Of High Blood Cholesterol In Adults (2001). Executive summary of the third report of the National Cholesterol Education Program (NCEP) expert panel on detection, evaluation, and treatment of high blood cholesterol in adults (adult treatment panel III). JAMA.

[24] American Diabetes Association Professional Practice Committee, ElSayed N.A., Aleppo G., Bannuru R.R., Bruemmer D., Collins B.S. (2024). 2. Diagnosis and classification of diabetes: standards of care in diabetes—2024. Diabetes Care.

[25] Chen Z., Cheang I., Zhu X., Qu Q., Chen S., Xing Y. (2025). Associations of body roundness index with cardiovascular disease and mortality among patients with metabolic syndrome. Diabetes Obes. Metab..

[26] Wang P., Fan Y., Gao H., Wang B. (2025). Body roundness index as a predictor of all-cause and cardiovascular mortality in patients with diabetes and prediabetes. Diabetes Res. Clin. Pract..

[27] Zhang X., Ma N., Lin Q., Chen K., Zheng F., Wu J. (2024). Body roundness index and all-cause mortality among US adults. JAMA Netw. Open.

[28] Huang B., Zhang X., Guo H., Meng C., Cui J., Jia J. (2025). U-shaped association of body roundness index with all-cause and cardiovascular mortality in individuals with chronic kidney disease. Ren. Fail..

[29] Liu H., Ye H., Zhang X., Wen Y., Wang J., Yu M. (2025). The association between body roundness index and mortality in diabetes. BMC Cardiovasc. Disord..

[30] Yi Y., Yang L. (2025). Association between body roundness index and risks of all-cause and cardiovascular mortality in adults with metabolic dysfunction-associated steatotic liver disease: NHANES 1999–2018. Front. Nutr..

[31] Thomas D.M., Bredlau C., Bosy-Westphal A., Mueller M., Shen W., Gallagher D. (2013). Relationships between body roundness with body fat and visceral adipose tissue emerging from a new geometrical model. Clin. Trials.

[32] Luo J., He Z., Li Q., Lv M., Cai Y., Ke W. (2023). Adipokines in atherosclerosis: unraveling complex roles. Front. Cardiovasc. Med..

[33] Liu L., Shi Z., Ji X., Zhang W., Luan J., Zahr T. (2022). Adipokines, adiposity, and atherosclerosis. Cell. Mol. Life Sci..

[34] Wang J., Wu M., Wu S., Tian Y. (2022). Relationship between body roundness index and the risk of heart failure in Chinese adults: the Kailuan cohort study. ESC Heart Fail..

[35] Zhang X., Ma N., Lin Q., Chen K., Zheng F., Wu J. (2024). Body roundness index and all-cause mortality among US adults. JAMA Netw. Open.

[36] Huang B., Zhang X., Guo H., Meng C., Cui J., Jia J. (2025). U-shaped association of body roundness index with all-cause and cardiovascular mortality in individuals with chronic kidney disease. Ren. Fail..

[37] Ziolkowski S.L., Long J., Baker J.F., Chertow G.M., Leonard M.B. (2019). Chronic kidney disease and the adiposity paradox: valid or confounded?. J. Ren. Nutr..

[38] Soohoo M., Streja E., Hsiung J.-T., Kovesdy C.P., Kalantar-Zadeh K., Arah O.A. (2022). Cohort study and bias analysis of the obesity paradox across stages of chronic kidney disease. J. Ren. Nutr..

[39] Chen R., Cai Y., Chen Q., Chen Z., Li H., Su Y. (2025). The association between the BRI and all-cause and cardiovascular mortality in COPD patients. BMC Pulm. Med..

[40] Huang Y., Maimaituxun A., Man Q., Liu S., Yang Y., Wen J. (2026). Visceral adiposity measured by Body Roundness Index linked to higher all-cause and cardiovascular mortality in osteoarthritis: results from NHANES 1999 to 2018. Med. (Baltimore).

[41] Jørgensen T.S.H., Osler M., Ängquist L.H., Zimmermann E., Christensen G.T., Sørensen T.I.A. (2016). The U-shaped association of body mass index with mortality: influence of the traits height, intelligence, and education. Obesity (Silver Spring).

[42] Piano M.R. (2017). Alcohol’s effects on the cardiovascular system. Alcohol Res..

[43] McMahan RH, Anton P, Coleman LG, Cresci GAM, Crews FT, Crotty KM, et al. Alcohol and immunology: mechanisms of multi-organ damage. Summary of the 2022 alcohol and immunology research interest group (AIRIG) meeting. Alcohol 2023;110:57–63. doi:10.1016/j.alcohol.2023.04.002. PMC10330898 37061143

[44] Seitz H.K., Moreira B., Neuman M.G. (2023). Pathogenesis of alcoholic fatty liver a narrative review. Life (Basel).

[45] Jayedi A., Soltani S., Zargar M.S., Khan T.A., Shab-Bidar S. (2020). Central fatness and risk of all cause mortality: systematic review and dose-response meta-analysis of 72 prospective cohort studies. BMJ.

[46] Krakauer N.Y., Krakauer J.C. (2014). Dynamic association of mortality hazard with body shape. PloS One.

[47] Ambrose J.A., Barua R.S. (2004). The pathophysiology of cigarette smoking and cardiovascular disease: an update. J. Am. Coll. Cardiol..

[48] Gustafson B., Hammarstedt A., Andersson C.X., Smith U. (2007). Inflamed adipose tissue: a culprit underlying the metabolic syndrome and atherosclerosis. Arterioscler. Thromb. Vasc. Biol..

[49] Celermajer D.S., Sorensen K.E., Georgakopoulos D., Bull C., Thomas O., Robinson J. (1993). Cigarette smoking is associated with dose-related and potentially reversible impairment of endothelium-dependent dilation in healthy young adults. Circulation.

[50] Chelland Campbell S., Moffatt R.J., Stamford B.A. (2008). Smoking and smoking cessation — the relationship between cardiovascular disease and lipoprotein metabolism: a review. Atherosclerosis.

