# Supplementary material for: Association of Body Roundness Index with all-cause and cardiovascular mortality in patients with cardiovascular-kidney-metabolic (CKM) syndrome stages 0–3: a cohort study
Source: Am Heart J Plus. 2026 Apr 4;65:100779. doi: 10.1016/j.ahjo.2026.100779 (PMC13091217; doi:10.1016/j.ahjo.2026.100779)
Supplement: Supplementary file 1 — Supplementary tables [file mmc1.docx]

# **Supplementary Material**

**Table S1. Definitions of CKM Syndrome Stages**

| **CKM syndrome stages** | **Definition** |
| --- | --- |
| Stage 0: No CKM Risk Factors. | Individuals with normal BMI and waist circumference, normoglycemia, normotension, a normal lipid profile, and no evidence of CKD or subclinical or clinical CVD.  (1)BMI < 25 kg/m2 (or < 23 kg/m2 if Asian ancestry).  (2)Waist circumference < 88/102 cm in women/men (or if Asian ancestry < 80/90 cm in women/men) |
| Stage 1: Excess or dysfunctional adiposity. | Individuals with overweight/obesity, abdominal obesity, or dysfunctional adipose tissue, without the presence of other metabolic risk factors or CKD.  (1)BMI ≥25 kg/m2 (or ≥23 kg/m2 if Asian ancestry).  (2)Waist circumference ≥88/102 cm in women/men (or if Asian ancestry ≥80/90 cm in women/men).  (3)Fasting blood glucose ≥100–124 mg/dL or HbA1c between 5.7% and 6.4%. |
| Stage 2: Metabolic risk factors and moderate- to high-risk CKD, or both. | Individuals with metabolic risk factors (hypertriglyceridemia, hypertension, MetS, diabetes), or moderate- to high-risk CKD  (1)Hypertriglyceridemia is defined by an TG of >135 mg/dL.  (2)Hypertension is defined by an SBP of ≥130 mm Hg, a DBP of ≥80 mm Hg, a medical diagnosis, or taking antihypertensive medication.  (3)MeTS is defined by the presence of ≥3 of the following: WC ≥ 102 cm for men or ≥ 88 cm for women; HDL-C <40 mg/dL for men, <50 mg/dL for women; TG ≥150 mg/dL; Elevated blood pressure (SBP ≥130 mm Hg, DBP ≥80 mm Hg, a medical diagnosis, or taking antihypertensive medication); FBG ≥100 mg/dL.  (4)Diabetes is defined by FBG levels of > 126 mg/dL, HbA1c levels of ≥ 6.5%, a medical diagnosis, or taking insulin or glucose-lowering medication.  (5) Moderate to high-risk CKD in the KDIGO classification is defined as UACR ≥ 30 mg/g and eGFR ≥ 60 ml/min/1.73m2, UACR < 300 mg/g and eGFR ≤ 45-59 ml/min/1.73m2, or UACR < 30 mg/g and eGFR ≤ 30-44 ml/min/1.73m2. |
| Stage 3: Subclinical CVD among individuals. | Subclinical CVD is defined as a high 10-year CVD risk or very high-risk CKD stage.  (1)A high 10-year CVD risk is defined as a 20% or above risk, as determined by the basic Predicting Risk of CVD EVENTs (PREVENT) equation.  (2)Very high-risk CKD in the KDIGO classification is defined as UACR ≥ 300 mg/g and eGFR ≤ 45-59 ml/min/1.73 m2, UACR ≥ 30 mg/g and eGFR ≤ 30-44 ml/min/1.73 m2, or eGFR ≤ 29 ml/min/1.73 m2. |
| Stage 4: Clinical CVD among individuals. | Clinical CVD (defined as self-reported diagnosis of cardiovascular disease, including heart failure, coronary heart disease, angina, myocardial infarction, or stroke) in individuals. |
| Abbreviations: CKM syndrome: Cardiovascular-Kidney-Metabolic syndrome; MeTS: metabolic syndrome; CKD: chronic kidney disease; CVD: cardiovascular disease; BMI: body mass index; WC: waist circumference; FBG: fasting blood glucose; KDIGO: Kidney Disease Improving Global Outcomes; UACR: urine albumin-to-creatinine ratio; eGFR: estimated glomerular filtration rate; TG: triglycerides; SBP: systolic blood pressure; DBP: diastolic blood pressure. | |

**Table S2 HR (95% *CI*) for all-cause and cardiovascular mortality according to BRI quintiles after excluding participants from the first 2 years of follow-up**

|  | HR (95% *CI*)，*P*-value | | |
| --- | --- | --- | --- |
|  | Model 1 | Model 2 | Model 3 |
| All-cause mortality |  |  |  |
| BRI | 1.10(1.07, 1.13), <0.001 | 1.05(1.01, 1.10), 0.016 | 1.05(1.00, 1.09), 0.038 |
| BRI (quintiles) |  |  |  |
| Q1 | 0.72(0.58, 0.89), 0.002 | 1.30(1.01, 1.67), 0.041 | 1.28(0.98, 1.67), 0.075 |
| Q2 | 0.92(0.73, 1.15), 0.448 | 1.13(0.90, 1.41), 0.306 | 1.10(0.88, 1.37), 0.416 |
| Q3 | 1 | 1 | 1 |
| Q4 | 1.38(1.10, 1.72), 0.005 | 1.25(1.01, 1.53), 0.039 | 1,20(0.96, 1.49), 0.108 |
| Q5 | 1.40(1.10, 1.78), 0.007 | 1.47(1.17, 1.83), <0.001 | 1.38(1.10, 1.73), 0.005 |
| *P*-trend | <0.001 | 0.051 | 0.124 |
| Cardiovascular mortality |  |  |  |
| BRI | 1.13(1.06, 1.20), <0.001 | 1.14(1.06, 1.21), <0.001 | 1.13(1.06, 1.21), <0.001 |
| BRI (quintiles) |  |  |  |
| Q1 | 0.93(0.59, 1.48), 0.770 | 1.00(0.66, 1.52), 0.995 | 1.02(0.68, 1.51), 0.940 |
| Q2 | 1.08(0.67, 1.73), 0.754 | 1.21(0.78, 1.87), 0.395 | 1.23(0.79, 1.92), 0.361 |
| Q3 | 1 | 1 | 1 |
| Q4 | 1.28(0.85, 1.93), 0.246 | 1.24(0.84, 1.84), 0.285 | 1,26(0.83, 1.89), 0.278 |
| Q5 | 1.95(1.32, 2.89), <0.001 | 2.05(1.41, 3.00), <0.001 | 2.05(1.39, 3.03), <0.001 |
| *P*-trend | <0.001 | <0.001 | 0.002 |
| ^a^ Model 1 was unadjusted.  ^b^ Model 2 was adjusted for age, gender, ethnicity, education, PIR.  ^c^ Model 3 was adjusted for age, gender, ethnicity, education, PIR, smoking, alcohol consumption, hypertension, hyperlipidemia, daily energy intake, and physical activity.  *BRI: Body roundness index, HR: Hazard ratio, 95%CI: 95% Confidence intervals. | | | |

**Table S3 SHR (95% *CI*) for cardiovascular mortality according to BRI quintiles after excluding participants from the first 2 years of follow-up**

|  | sHR (95% *CI*)，*P*-value | | |
| --- | --- | --- | --- |
|  | Model 1 | Model 2 | Model 3 |
| Cardiovascular mortality |  |  |  |
| BRI | 1.10(1.07, 1.14), <0.001 | 1.08(1.03, 1.14), 0.002 | 1.08(1.03, 1.14), 0.002 |
| BRI (quintiles) |  |  |  |
| Q1 | 0.74(0.52, 1.05), 0.092 | 1.21(0.85, 1.73), 0.281 | 1.16(0.81, 1.66), 0.940 |
| Q2 | 1.03(0.75, 1.42), 0.849 | 1.21(0.88, 1.67), 0.230 | 1.20(0.87, 1.64), 0.265 |
| Q3 | 1 | 1 | 1 |
| Q4 | 1.52(1.13, 2.03), 0.005 | 1.31(0.98, 1.75), 0.068 | 1,29(0.96, 1.73), 0.087 |
| Q5 | 1.56(1.16, 2.09), 0.003 | 1.69(1.26, 2.28), <0.001 | 1.62(1.20, 2.19), 0.002 |
| *P*-trend | <0.001 | 0.009 | 0.012 |
| ^a^ Model 1 was unadjusted.  ^b^ Model 2 was adjusted for age, gender, ethnicity, education, PIR.  ^c^ Model 3 was adjusted for age, gender, ethnicity, education, PIR, smoking, alcohol consumption, hypertension, hyperlipidemia, daily energy intake, and physical activity.  *BRI: Body roundness index, sHR: subdistribution Hazard ratio, 95%CI: 95% Confidence intervals. | | | |
